# Reversible Renal Insufficiency Secondary to Extrinsic Splenic Compression of the Kidney in a Patient with Chronic Lymphocytic Leukemia

**DOI:** 10.1100/tsw.2010.74

**Published:** 2010-05-04

**Authors:** Miriam Hadj-Moussa, James A. Brown

**Affiliations:** Division of Urology, Medical College of Georgia, Augusta, GA, USA

**Keywords:** renal insufficiency, splenomegaly, leukemia, lymphocytic, chronic

## Abstract

While increased renal venous and direct renal parenchymal pressure may cause renal insufficiency, there are no prior reports of hypersplenism secondary to chronic lymphocytic leukemia (CLL) doing so. This first report of massive splenomegaly leading to marked compression of the left kidney associated with renal insufficiency that resolved after splenectomy illustrates that profound extrinsic renal compression from splenomegaly may significantly compromise left renal function and splenectomy should be considered in this situation.

## INTRODUCTION

The mechanism through which increased intra-abdominal pressure causes renal insufficiency is likely multifactorial, and includes both increased renal venous pressure and direct renal parenchymal pressure[1,2].

Chronic lymphocytic leukemia (CLL) is frequently associated with hypersplenism and massive splenomegaly. A Medline review of the literature from 1966 to 2009 did not identify any reports of reversible renal insufficiency secondary to extrinsic splenic compression of the left kidney. We report a case of renal insufficiency that resolved after splenectomy in a CLL patient with marked compression of the left kidney due to massive splenomegaly.

## CASE REPORT

A 77-year-old male with a history of CLL presented for treatment for hypersplenism with thrombocytopenia. A CT scan (Fig. 1) showed massive splenomegaly, bulky chronic lymphadenopathy, an enhancing 2.8-cm right midpole renal mass, and a markedly compressed left kidney. A Lasix MAG 3 renal scan demonstrated decreased left renal function (24%) with no evidence of obstruction. A small hypervascular right renal mass, and normal bilateral renal arteries and veins without obstruction, were visible on renal arteriogram. Subsequently, the patient underwent splenectomy, cholecystectomy, and right partial nephrectomy for grade 2, stage T1, clear cell, renal cell carcinoma.

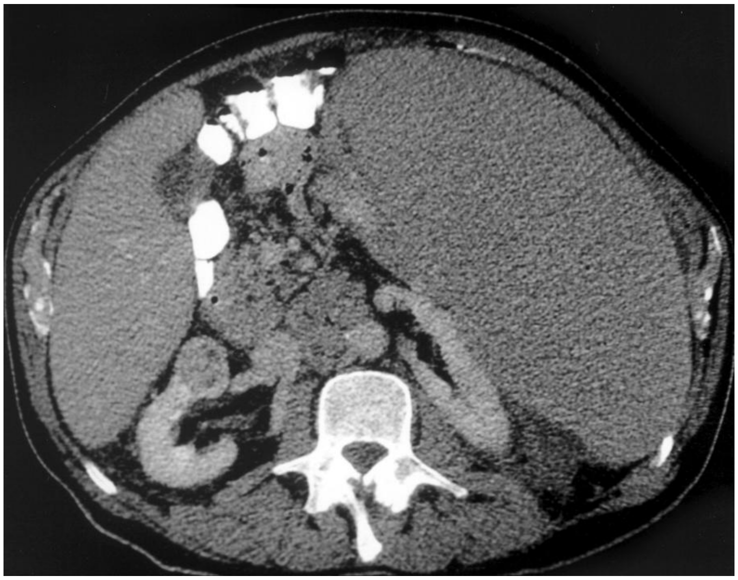

**FIGURE 1.** Preoperative abdominal CT scan demonstrating marked left renal compression secondary to massive splenomegaly and right renal tumor.

One month postoperatively, the patient underwent a Lasix DTPA renal scan, which revealed 52% left and 48% right relative differential renal function, no obstruction, and an improvement in the time-to-peak activity of the left kidney from 8 to 6 min. A 6-month postoperative abdominal CT scan showed a left kidney with normal reniform appearance, and a right kidney with dystrophic calcification and no tumor recurrence (Fig. 2). A Lasix MAG 3 renal scan of the left kidney at 1 year demonstrated 60% relative function, as well as improved renal perfusion and renal uptake compared to the preoperative Lasix MAG 3 renal scan. Twelve-, 18-, and 30-month postoperative renal CT scans demonstrated stable kidneys and lymphadenopathy. The patient's baseline serum creatinine was unknown, but preoperative serum creatinine was 1.4 mg/dL and improved to 1.2 mg/dL at 3 months and 3 years postoperatively. No other prerenal, renal, or postrenal causes for the elevated serum creatinine were identified by the patient's internist or urologist. Additionally, no other explanation, other than splenectomy and decreased renal compression, was found to explain the improved postoperative serum creatinine.

## DISCUSSION

Increased intra-abdominal pressure associated with laparoscopic surgery results in a decrease in urinary output from transient renal insufficiency that returns to normal within several hours after surgery[2]. Porcine studies have demonstrated that increasing renal venous pressure by 30 mmHg for 2 h results in decreased renal artery blood flow and glomerular filtration rate with increased plasma renin, serum aldosterone, and urinary protein leak[3,4]. Increased renal parenchymal pressure alone has not been conclusively shown to produce renal dysfunction, but Doty and colleagues suggest it may worsen the effects of increased renal venous pressure[3,4]. Preoperative renal angiography did not show an obstructed left renal vein; therefore, the etiology of this patient's left renal insufficiency was apparently direct renal parenchymal pressure with or without partial renal vein obstruction secondary to massive splenomegaly.

This case illustrates that profound extrinsic renal compression from splenomegaly may significantly compromise left renal function and splenectomy should be considered in these cases.

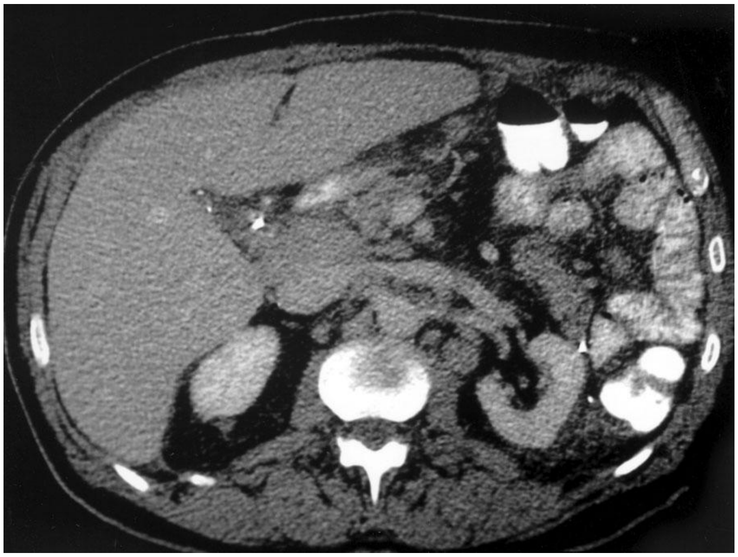

**FIGURE 2.** Six-month postoperative abdominal CT scan demonstrating a left kidney with normal appearance after splenectomy.
